# Ultrasound technology and tomato industry: A review

**DOI:** 10.1016/j.ultsonch.2025.107374

**Published:** 2025-05-02

**Authors:** Amir Shafaei Fallah, Fateme Asadi Touranlou, Mitra Rezaie

**Affiliations:** Department of Nutrition, Faculty of Medicine, Mashhad University of Medical Sciences, Mashhad, Iran

**Keywords:** Ultrasound, Agrochemicals, Bio-compound, Extraction, Germination, Sonication, Tomato

## Abstract

Recent research emphasizes the potential of ultrasound technology in the food industry, particularly in tomato processing. Ultrasound’s non-invasive nature has made it a preferred choice for researchers seeking extraction and cavitation power without significantly altering sample properties. The concept of waste valorization, which involves transforming waste materials into valuable resources, is gaining traction worldwide as it helps reduce carbon footprints and pollution. Tomato processing yields significant waste, such as peels and seeds, which contain high levels of antioxidants and fiber, making them valuable resources for the food industry. This review examines the diverse applications of ultrasound technology in the tomato industry, focusing on its effects on the extraction of bioactive compounds, inhibition of microbial growth, enhancement of tomato germination, and removal of agrochemicals. Findings show that ultrasound-assisted extraction (UAE) increases yields of pectin and lycopene by 34–77% compared to conventional methods, reduces pesticide residues by up to 97%, and enhances germination rates by up to 43% when combined with technologies like plasma-activated water or ozone. Ultrasound significantly reduces bacteria, phytoviruses, yeasts, and molds, with reductions of 1.5–3.4 log CFU/g in tomato products. However, ultrasound treatment may affect seedling vigor due to reactive oxygen species generation. Further research is needed to optimize ultrasound parameters for diverse crops and integrate it with emerging technologies to maximize industrial scalability.

## 1 Introduction

Tomato (Solanum Lycopersicon L.) is consumed both fresh and in processed forms. It is a key ingredient in various food products, from condiments like ketchup and paste to sauces and juices [1]. Global tomato production exceeds 186 million tons annually, with over 130 million tons processed, generating approximately eight million tons of waste, including peels (61 %) and seeds (38 %) [[2], [3], [4]].

Manufacturers are repurposing the residual byproducts from the manufacturing process as input materials for the extraction of beneficial bioactive agents, including lycopene and phenolic compounds [5]. To promote food waste reduction and nutrient recovery, the circular economy action plan encourages the use of byproducts like tomato peels and seeds, which have high concentrations of polyphenols [6]. However, the tomato processing industry faces environmental and economic challenges due to waste generation, including greenhouse gas emissions and water waste.

To address these challenges, innovative technologies such as sonication are emerging in the food processing industry. This eco-friendly and sustainable technology offers a low-energy, high-efficiency alternative to conventional decontamination processes while minimizing secondary pollutants [7]. Ultrasound technology has various applications in food processing, providing unique physical and chemical advantages over traditional methods, which include enhanced processing times, improved product quality, reduced risks, and greater environmental sustainability [8].

**Fig. 1:**
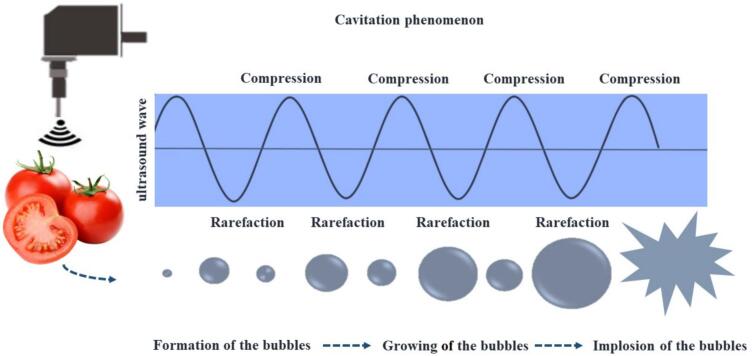
Mechanism of ultrasound cavitation.

Ultrasound-assisted extraction (UAE) is an advanced technique that provides a more efficient method for isolating bioactive agents due to its significant advantages, including shorter processing times, improved yields, and reduced energy and solvent consumption [1]. UAE operates on the principle of cavitation, which involves the formation, expansion, and subsequent implosion of bubbles within the extraction solvent and the sample material [9] (See Fig. 1). Beyond extraction, ultrasound provides additional benefits in food processing, including microbial inactivation, agrochemical degradation, and improved seed germination, all of which enhance product safety and agricultural outcomes [10,11].

Despite extensive research on ultrasound applications in food preservation and processing, no comprehensive review has specifically explored its role in the tomato industry. This article synthesizes current knowledge on ultrasound’s diverse applications in tomato processing and cultivation, focusing on four key areas: (1) extraction of bioactive compounds from tomato byproducts, (2) enhancement of seed germination and seedling vigor, (3) degradation of fungicide residues, and (4) improvement of microbial stability. By transforming tomato waste into a resource for high-value compounds, ultrasound supports a waste-to-wealth strategy while addressing critical food safety and sustainability concerns. This review aims to provide researchers, industry professionals, and stakeholders with a robust resource to advance innovative solutions in tomato processing and cultivation.

## 2 Bioactive compound extraction

Tomatoes are a rich source of compounds with various health-promoting properties, including antioxidant, anti-mutagenic, anti-proliferative, anti-inflammatory, and anti-atherogenic activities. These compounds include vitamins such as ascorbic acid and vitamin A, a range of phenolic compounds like phenolic acids and flavonoids, carotenoids such as lycopene, α-carotene, and β-carotene, as well as glycoalkaloids, notably tomatine [12].

**Fig. 2:**
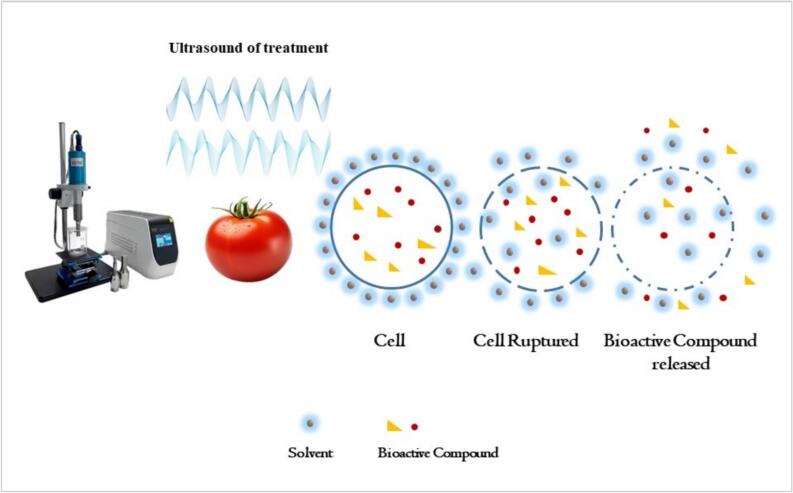
Extraction of bioactive compounds using ultrasound-assisted extraction.

Ultrasound-assisted extraction (UAE), utilizing high-frequency sound waves (>20 kHz), enhances the extraction of these compounds by disrupting cell matrices through cavitation, improving solvent penetration and mass transfer (see Fig. 2). Table 1 summarizes key findings from relevant studies.

**Table 1:** Bioactive compound extraction efficiency of tomato using Ultrasound.

*Author & Year*	*Country*	*Parts of the tomato*	*Target compound*	*Operating condition*	*Major Findings*	*Ref*
Grassino et al. 2015	Italy	Tomato Waste	Pectin	37 kHz 60 & 80 ˚C	34–36 % yield in 15 min vs. 1440 min for conventional extraction	[13]
Sengkhamparn et al. 2019	Thailand	Tomato Waste	Pectin	80 ˚C 20 min	9.8 % yield, enhanced by low pH	[14]
Sengar et al. 2019	India	Tomato Waste	Pectin	20 KHz 60 ˚C 450, 600 W and 750 W.	15.21 % yield, 73.33 % esterification	[15]
Marinaccio et al. 2024	Turkey	tomato skin waste	Lycopene	20 kHz 400 W 20 min	735.9 mg/g yield using n-hexane	[18]
Kuvendziev et al. 2024	Slovenia	Tomato skins waste	Lycopene	40 kHz for 120 min	Maximizing lycopene yield, which ranges from 1018 to 1120 mg/kg.	[19]
Yilmaz et al. 2021	Turkey	tomato-processing wastes	Lycopene and β-carotene	90 W 15 and 30 min	The highest yields of lycopene and β-carotene were achieved with 90 W ultrasonic power for 30 min and 15 min, respectively.	[26]
Liao et al. 2016	China	Tomatoes	Lycopene	200 W 46–48 kHz	The IUAE ^b^ method achieved a substantial increase in extraction yield, a notable reduction in extraction time, and a decreased requirement for solvent at lower temperatures.	[34]
Amiri-Rigi et al. 2016	Tehran	tomato industrial waste	Lycopene	30 to 480 s 50 W	33.4 ± 3.95 % yield, reduced by prolonged sonication	[25]
Teixeira et al. 2024	Brazil	purple tomatoes	Anthocyanins	5 to 75 min	54–73 % higher yield vs. conventional	[22]
Mozafari et al. 2024	Spain	tomato pomace	Lycopene and β-carotene	700 W 20 kHz 15–25 min	The highest lycopene extraction was achieved at 45 °C with 82 % amplitude over 28 min, while the maximum Σβ-carotene extraction occurred under the same temperature and amplitude conditions in 23 min.	[23]
Altunay et al. 2022	Turkey	tomato	β-carotene	5 min	The recovery of β-carotene was observed to be an impressive 98.3 %.	(20)
Prokopo et al. 2016	Bulgaria	tomato peels	Carotenoid extraction	45 kHz 5, 10, 15 and 20 min	23.9–28.2 % higher yield vs. conventional	[21]
Solaberrieta et al. 2022	Spain	Tomato seed wastes	Antioxidants	23.9 kHz 15 min, 80 ◦C	The optimal parameters for UAE were determined to be an extraction time of 15 min, a solvent concentration of 61 % ethanol, and an amplitude of 85 %.	[27]
Li et al. 2022	Italy	Tomato Waste	Lycopene	20 kHz 35–65 ◦C 20–50 min	Ultrasound-assisted extraction offers an improvement in the yield of extracted bioactive compounds, a reduction in the amount of solvent required, and a decrease in the time required for the extraction process.	[16]
Jose et al. 2018	Brazil	cherry tomato	Lycopene or β-carotene	30 min	Following a storage period of 10 days at a temperature of 7 °C, it was observed that the content of both lycopene and β-carotene in the extracted samples remained stable.	[35]

^a^ Ultrasound-assisted extraction, ^b^ improved ultrasonic-assisted extraction

Grassino et al. [13] employed UAE to extract pectin from tomato canning waste using a 37 kHz ultrasound bath at 60–80 °C, achieving a 34–36 % yield in just 15 min, compared to 1440 min for conventional solvent extraction. The extracted pectin was purified through ethanol precipitation, centrifugation, and drying to remove residual sugars and proteins, ensuring compliance with food-grade standards [13]. Sengkhamparn et al. [14] optimized UAE for pectin from tomato paste processing waste, reporting a 9.8 % yield at 80 °C, 20 min, and pH 2.0 (citric acid solution). Higher temperatures and longer durations increased yields due to enhanced pectin solubility. Purification involved ethanol precipitation, filtration, and drying to eliminate co-extracted compounds [14]. The authors suggested lower pH could further improve yields. Senger et al. [15] extracted pectin from tomato peel waste using UAE at 20 kHz, 600 W, and 60 °C for 8.61 min, yielding 15.21 %. Post-extraction purification involved ethanol precipitation, washing, and lyophilization to remove residual solvents and impurities. However, higher power levels led to pectin degradation into monosaccharides, underscoring the importance of parameter optimization. Li et al. [16] utilized UAE with ethanol to recover lycopene from tomato waste. The optimized conditions reported in as T = 65 °C, t = 20 min, yielding 1536 ± 53 µg/g of lycopene.

Yadav et al. [17] developed integrated ultrasound-surfactant assisted extraction (IUSAE) for lycopene extraction from tomato peels, achieving a 77 % yield in 30–90 % less time than conventional solvent-assisted extraction (SAE) or standalone UAE. IUSAE required 30 % less surfactant and 85 % less time than two-step UAE, reducing environmental impact [17]. Marinaccio et al. [18] used UAE (20 kHz, 400 W) with deep eutectic solvents and n-hexane to extract 735.9 mg/g lycopene from tomato skin, with pre-treatments like drying and grinding enhancing yields by reducing particle size and mass transfer resistance. Kuvendziev et al. [19] combined enzymatic pre-treatment with UAE (40 kHz, 15 min), yielding up to 1120 mg/kg lycopene, purified through filtration and solvent removal.

Ultrasound has been demonstrated to be an effective method for extracting carotenoids from tomato by-products, such as skin, seeds, and some pulp. In a study conducted by Luengo et al. [20], the combination of ultrasound with moderate pressure (100 kPa) resulted in an increased carotenoid extraction yield of 17.37 mg per 100 g of dry weight (DW). This represents a 149 % improvement over traditional methods, without causing any degradation of the carotenoids. Prokopov et al. [21] demonstrated that UAE (45 kHz, 5–10 min) increased carotenoid and β-carotene yields by 23.9–28.2 % in Bulgarian tomato peels compared to conventional methods. In the Stella cultivar, 5-minute UAE boosted total carotenoids and β-carotene by 27.2 ± 1.1 % and 28.2 ± 0.1 %, respectively, while the Karobeta cultivar showed 23.9 ± 2.4 % and 26.5 ± 1.0 % increases after 10 min [21]. Teixeira et al. [22] reported 54–73 % higher anthocyanin yields in purple tomatoes using UAE (25 kHz, 50 % amplitude). Mozafari et al. [23] optimized UAE for lycopene and β-carotene, while Altunay et al. [24] recovered 25.9 ± 1.7 μg kg^−1^ of β-carotene using ultrasound-assisted liquid–liquid micro-extraction with phase separation. Amiri-Rigi et al. [25] achieved a 33.4 ± 3.95 % lycopene yield using UAE with microemulsion (20 kHz, 50 W, 3 min) but noted that prolonged sonication reduced yields due to enzyme release (e.g., lipoxygenase) from excessive cell disruption. Yilmaz et al. [26] optimized UAE for lycopene and β-carotene from tomato waste (24 kHz, 90 W, 15–30 min), reporting yields of 73.73–76.87 mg/kg lycopene.

Senger et al. [15] also explored ultrasound assisted microwave extraction (UAME), which yielded pectin with a 73.33 ± 1.76 % degree of esterification, high galacturonic acid content, and lighter color, purified via ethanol precipitation and lyophilization. Solaberrieta et al. [27] applied UAE to tomato seed waste, optimizing conditions to maximize total phenolic content (TPC) and antioxidant activity (DPPH) using response surface methodology, highlighting UAE’s efficacy in valorizing seed byproducts.

### 2.1 Comparative analysis of ultrasound-based extraction methods

Ultrasound-based extraction methods, such as UAE, UAME, and IUSAE, leverage the sonochemical effects of cavitation to enhance the recovery of bioactive compounds from tomato byproducts. However, their performance varies in terms of extraction efficiency, resource demands, product quality, scalability, environmental sustainability, and regulatory alignment, making each method suited to specific industrial contexts.

UAE is the most widely studied and versatile method, achieving high yields for pectin (34–36 % [13], 15.21 % [15]), lycopene (735.9 mg/g [18], 1120 mg/kg [19]), and other compounds in 5–20 min, compared to hours for conventional solvent extraction, which relies on prolonged heating and large solvent volumes [13]. The cavitation process generates localized high-pressure and temperature zones, disrupting cell walls and enhancing solvent diffusion, which reduces processing time and resource use [28]. Its simpler ultrasonic baths or flow-through reactors require lower capital investment than the complex reactors, distillation units, and solvent recovery systems of conventional methods, making it accessible for both small and large facilities [29]. Operationally, UAE reduces energy and solvent consumption compared to conventional extraction’s high energy demands for heating and solvent recovery, with shorter treatment times further lowering costs [28,30]. However, excessive sonication can degrade heat-sensitive compounds like lycopene, necessitating precise parameter control to avoid enzymatic or oxidative losses [15,24].

UAME combines ultrasound with microwave energy to enhance cell disruption through synergistic thermal and mechanical effects, producing pectin with superior quality attributes, such as a 73.33 ± 1.76 % degree of esterification and high galacturonic acid content [15]. These properties make UAME ideal for premium applications, such as functional foods or pharmaceuticals, where pectin’s gelling and stabilizing capabilities are critical [31]. However, UAME’s specialized reactors and higher energy demands increase capital and operational costs compared to UAE, and the thermal component of microwaves may exacerbate degradation risks for thermolabile compounds, requiring careful optimization [28]. While solvent use remains 50–70 % lower than conventional methods, UAME’s complexity limits scalability, making it less suitable for high-throughput tomato processing compared to UAE [15].

IUSAE integrates ultrasound with surfactants to enhance lycopene extraction, achieving a 77 % yield in 30–90 % less time than SAE or standalone UAE, with 30 % less surfactant and 85 % less processing time than two-step UAE [17]. The surfactant reduces surface tension, improving cavitation efficiency and compound solubilization, which minimizes environmental impact through reduced resource use [32]. However, the presence of surfactants necessitates rigorous purification (e.g., filtration, solvent removal) to eliminate residues [17]. IUSAE’s energy consumption is comparable to UAE, but its scalability is constrained by purification challenges, limiting its adoption to facilities focused on high-value lycopene production [17].

Conventional solvent extraction, while effective for pectin, carotenoids, and phenolics, requires complex infrastructure and extensive solvent use, leading to high capital and operational costs due to prolonged processing times and energy-intensive heating and solvent recovery [30,31]. It also demands extensive purification to remove solvent residues, increasing costs and environmental impact, particularly for food-grade applications [28]. In contrast, ultrasound methods preserve compound quality by operating non-thermally or with minimal heat, reducing the need for additional purification or reformulation and enhancing market value for premium products [15,30].

UAE’s scalability is a key advantage, with continuous-flow systems capable of processing large volumes of tomato byproducts, facilitating faster return on investment, especially for facilities prioritizing sustainability [31]. UAME, while producing high-quality pectin, is better suited to niche markets requiring specialized products, as its higher costs and complexity hinder widespread adoption [15]. IUSAE offers advantages for lycopene extraction in facilities prioritizing rapid processing, but its purification requirements and chemical use limit its versatility compared to UAE [17]. Environmental sustainability further favors UAE, as its lower resource demands and absence of chemical additives align with circular economy goals, potentially qualifying for environmental subsidies [28].

From an industrial perspective, UAE is the most cost-effective and scalable, enabling integration into existing processing lines for tomato canning and paste production, where throughput and cost efficiency are critical [30]. Its broad applicability across pectin, carotenoids, and phenolics, coupled with regulatory acceptance, makes it the preferred choice [21,30]. UAME suits high-value markets requiring specialized pectin, but its costs hinder widespread adoption [33]. IUSAE offers advantages for rapid lycopene extraction, but its chemical use and purification needs limit its versatility. Compared to conventional solvent extraction, ultrasound methods provide higher yields, lower costs, and better quality retention, positioning them as transformative for tomato byproduct valorization [28]. Future research should focus on mitigating UAME’s energy demands through advanced reactor designs, optimizing IUSAE’s surfactant formulations to reduce purification needs, and standardizing UAE parameters across tomato varieties to enhance consistency and industrial applicability [31].

## 3 Seed germination and seedling performance

**Fig. 3:**
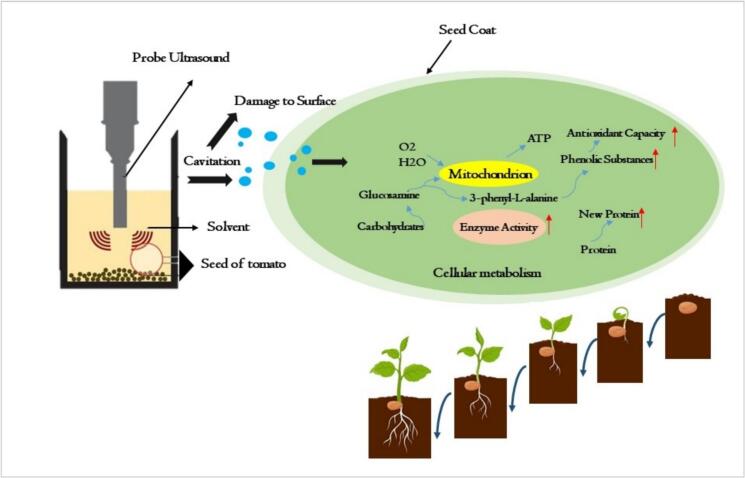
Ultrasound impact on germination and seeding of tomato.

Seed quality is a cornerstone of agricultural productivity, with approximately 95 % of cultivated crops, including tomatoes, propagated through seeds [36]. Enhancing seed germination and seedling vigor is critical for ensuring robust crop establishment and yield. Ultrasound technology has emerged as a promising physical treatment to break seed dormancy, stimulate metabolic activity, and improve germination rates [37] (See Fig. 3). This section reviews the application of ultrasound-based treatments, including standalone ultrasound, ultrasound combined with ultraviolet-C (UV-C), and ultrasound with plasma-activated water (PAW), in enhancing tomato seed germination and seedling performance. A comparative analysis evaluates their efficacy, scalability, and suitability for industrial adoption, with key findings summarized in Table 2.

**Table 2:** Germination and seeding rate of tomato using Ultrasound.

Author & Year	Country	Type of Processing	Operating condition	Major Findings	Ref
Nogueira et al. 2023	Portugal	US	19.8 kHz 200 W	43 % germination increase, 19 % faster	[38]
Ramteke et al. 2015	India	US	40 kHz 100 W	US ^a^ improved germination, root length, and seedling height	[41]
Baser Kouchebagh et al. 2015	Iran	US	10 min	Ultrasound seeds had a higher amount of leaf area index of 870 %, number of fruits per plant by 40 %, fruit yield (kg ha-1) by 46.4 %, and above-ground mass by 1.2 % than control	[42]
Fernandes et al. 2020	Brazil	US + gas exchange	0, 3, 6, and 9 s	Duration of sonication affected the number of shoots over 0.5 cm and leaflets, indicating an interaction with sealing type and emphasizing its impact on morphogenesis.	[43]
Kibar et al. 2020	Turkey	US + Ultraviolet-C	35 KHz	UV-C + US ^b^ treatment increased seedling height, seedling fresh weight, and seedling dry weight by 16.99–45.11 %, 24.82–39.88 %, and 37.93––52.63 %.	[40]
MEMİŞ et al. 2022	Turkey	US	50 kHz 30 min	The positive effects of ultrasound treatment on seedling emergence were a significant 13 % increase in seedling emergence rates compared to the control samples.	[39]

^a^: Ultrasound, ^b^: Ultraviolet-C + Ultrasound.

Nogueira et al. [38] investigated the effects of a 19.8 kHz, 200 W ultrasonic prototype on tomato, turnip, maize, and watermelon seeds. For tomato seeds, ultrasound treatment increased germination by 43 %, reduced mean germination time by 19 %, and decreased mean emergence time by 10 %, demonstrating significant improvements in seedling development. These outcomes suggest ultrasound’s potential to enhance agricultural productivity [38]. Memiş et al. [39] reported a 12 % increase in tomato seedling emergence using 50 kHz ultrasound for 30 min, attributing the enhancement to cavitation-induced microvoids that improve water uptake and stimulate germination.

Kibar [40] explored the combined effects of ultrasound (35 kHz) and UV-C (200–280 nm) on tomato seedling growth, observing increases in seedling height (16.99–45.11 %), fresh weight (24.82–39.88 %), and dry weight (37.93–52.63 %) across four varieties compared to untreated controls. The synergistic action of ultrasound’s mechanical disruption and UV-C’s photochemical effects enhanced vigor more than either treatment alone [40]. Ramteke et al. [41] treated tomato seeds in a 40 kHz, 100 W ultrasonic bath, recording higher germination percentages, survival rates, and seedling heights after 2, 4, and 10 days compared to controls soaked in distilled water.

Baser Kouchebagh et al. [42] found that ultrasound-treated tomato seeds exhibited an 870 % increase in leaf area index, 40 % more fruits per plant, 46.4 % higher fruit yield (kg ha^−1^), and 1.2 % greater above-ground mass, though chlorophyll content decreased by 30.2 %. Fernandes et al. [43] demonstrated ultrasound’s role in promoting organ development in vitro, attributing effects to cavitation-induced membrane rupture and subsequent repair, which enhances substance absorption without permanent cellular damage.

### 3.1 Comparative analysis of ultrasound-based seed treatments

Standalone ultrasound, ultrasound with UV-C, ultrasound with PAW, and conventional hydropriming each offer distinct approaches to enhancing tomato seed germination and seedling performance, with differences in efficacy, mechanisms, scalability, and costs shaping their suitability for industrial applications. Standalone ultrasound significantly improves germination rates (up to 43 % increase [38]) and seedling vigor (e.g., 12 % higher emergence [39], increased seedling height and survival [41]) by inducing cavitation that creates microvoids in seed coats, enhancing water uptake and stimulating metabolic activity [37]. Its non-chemical, non-thermal nature ensures no residue risks, making it ideal for organic seed production [38]. Ultrasound with UV-C enhances vigor further, increasing seedling height (16.99–45.11 %), fresh weight (24.82–39.88 %), and dry weight (37.93–52.63 %) through synergistic mechanical (cavitation) and photochemical (UV-C-induced stress responses) effects, making it suitable for high-value varieties requiring robust seedlings [40]. Ultrasound with PAW leverages reactive oxygen species (ROS) generated in PAW’s reactive environment to break dormancy, particularly in recalcitrant seeds, but its efficacy depends on controlled conditions to balance stimulatory ROS effects with potential oxidative damage [44,45]. Excessive ROS generated during ultrasonic treatment of PAW-soaked seeds may lead to oxidative stress, causing lipid peroxidation, protein denaturation, or DNA damage, which could negatively impact seed metabolism and seedling vigor [46]. This risk is particularly pronounced in seeds with low antioxidant capacity or under prolonged exposure to high ROS levels. Implementing controlled ultrasound conditions can minimize these risks and note the need for further research to quantify ROS effects. Conventional hydropriming, a standard seed treatment, enhances germination through controlled water imbibition but requires longer soaking times (hours to days) and precise moisture control, limiting its efficiency compared to ultrasound methods [47].

From an economic perspective, conventional hydropriming requires a low capital investment due to the simplicity of the soaking equipment. However, it incurs high operational costs because of prolonged treatment durations and substantial water usage. Standalone ultrasound requires moderate capital investment for ultrasonic baths or flow-through systems, which are less costly than complex hydropriming setups with drying units, and its short treatment times (5–30 min) and low energy requirements minimize operational costs, making it highly cost-efficient [38,39]. Ultrasound with UV-C demands higher capital investment for integrated ultrasound-UV-C systems and increased operational costs due to UV-C energy demands and maintenance, limiting its cost-effectiveness for large-scale applications [40]. Ultrasound with PAW requires specialized PAW generation equipment, significantly increasing capital costs and its operational costs are elevated by energy demands and the need for precise ROS management, reducing its economic viability for broad adoption [48].

Quality preservation is a key differentiator, as hydropriming’s prolonged soaking can lead to uneven germination or microbial contamination, requiring chemical treatments that increase costs and regulatory scrutiny [47]. Standalone ultrasound preserves seed quality by avoiding chemical inputs minimizing treatment time, reducing contamination risks, and aligning with organic standards [38]. Ultrasound with UV-C enhances seedling quality but risks UV-induced damage to seed DNA if not carefully controlled, necessitating optimization [49]. Scalability favors standalone ultrasound, as its continuous-flow systems can treat large seed volumes, facilitating rapid integration into existing seed processing workflows, with pilot trials in maize and rice indicating transferability to tomatoes [47]. Ultrasound with UV-C and PAW are less scalable due to equipment complexity and precise control requirements, making them better suited for niche or research applications [48,49]. Conventional hydropriming, while scalable, is less efficient due to longer processing times and drying needs [47].

Environmental sustainability strongly favors standalone ultrasound, as it eliminates chemical inputs and reduces water use compared to hydropriming, aligning with sustainable agriculture goals and potentially qualifying for environmental subsidies [28]. Ultrasound with UV-C and PAW increases energy demands and system complexity, potentially offsetting environmental benefits [48,49]. UV-C and PAW methods require validation to ensure no adverse effects on seed quality or safety, posing potential hurdles for commercial adoption. From an industrial perspective, standalone ultrasound is the most viable due to its low cost, scalability, and regulatory alignment, making it ideal for commercial seed treatment facilities [49]. Ultrasound with UV-C is advantageous for niche applications requiring enhanced vigor, while ultrasound with PAW suits research or crops with high dormancy barriers but requires optimization to compete with simpler methods [48,49]. Compared to hydropriming, ultrasound methods offer superior efficacy, lower costs, and better quality retention, positioning them as transformative for tomato seed treatment [28]. Future research should focus on standardizing ultrasound parameters across tomato varieties, quantifying ROS impacts in PAW treatments, and developing cost-effective UV-C systems to enhance the competitiveness of combined methods [48,49].

## 4 Agrochemical degradation

**Fig. 4:**
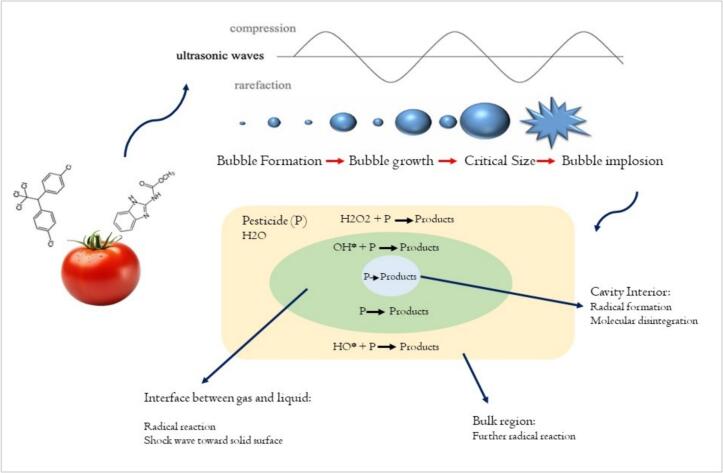
Ultrasound effect on degradation agrochemicals in tomato.

Pesticide residues on fresh produce, such as tomatoes, pose significant food safety and environmental challenges, traditionally mitigated through washing, cooking, chemical treatments, or heat processing. Ultrasound technology has emerged as an innovative, non-thermal method for degrading agrochemical residues. It offers several advantages over conventional approaches and competing advanced oxidation processes, such as ozone treatment and the Fenton process (Fig. 4).

This section reviews the application of ultrasound-based methods, including standalone ultrasound, ultrasound with vinegar or hydrogen peroxide, and ultrasound combined with PAW or ozone, in reducing pesticide residues on tomatoes. While ultrasound shows promise, standardized protocols for ultrasonic pesticide removal are still evolving, and there are currently no universally adopted guidelines specific to this method. However, general food safety standards, such as the Codex Alimentarius, provide guidance on residue limits [50].

The impact of ultrasound processing on the degradation of agrochemicals is presented in Table 3.

**Table 3:** Degradation agrochemicals in tomato using Ultrasound.

Author & Year	Country	Type of agrochemicals	Type of Processing	Operating condition	Major Findings	Ref
Ali et al. 2022	China	chlorothalonil fungicide residues	US a + plasma activated liquid	50 kHz 1,3,5,10 min	The highest chlorothalonil fungicide residues were reduced by 89.28 and 80.23 % for plasma-activated water-US and plasma-activated buffer solution-US.	[51]
Hosny Ahmed et al. 2024	Egypt	Pesticide Residues	US + 4 % vinegar or 3 % salt	10 min	4 % vinegar with sonication can lead to higher pesticide reduction levels for certain pesticides compared to the treatment without sonication	[52]
Manzoor et al. 2023	China	anilazine fungicide	US	40 kHz, 480 W 40 min	Ultrasound treatment can successfully remove up to 80.52 % of the anilazine fungicide after 40 min of application.	[9]
Tan et al. 2024	China	dimethoate, dichlorvos, methiocarb, propoxur, chlorobenzuron, and etridiazole,	US + H_2_O_2_	40 kHz	The combination of hydrogen peroxide and ultrasonic treatment (H2O2/US process) was observed to achieve exceptional efficacy in the removal of pesticide residues, reaching high rates of removal for the following pesticides: dimethoate (83.9 %), dichlorvos (68.8 %), methiocarb (85.9 %), propoxur (91.6 %), chlorobenzuron (75.6 %), and etridiazole (73.4 %).	[53]
Yang et al. 2024	China	pesticide residues	US + O3	20 to 40 kHz	The US/O3 process was found to be highly effective in eliminating pesticide residues, with impressive removal rates ranging from a minimum of 79.1 % to a maximum of 92.2 %	[54]
Ali et al. 2023	China	fungicide	US + plasma activated liquid	15 min	a significant reduction of pesticide residues to the extent of 97.25 % was achieved by plasma-activated water −U_10_, followed by 93.14 % in plasma-activated buffer solution −U_10_.	[10]

a : Ultrasound.

Ali et al. [51] conducted a study on the synergistic effects of combining plasma-activated liquid (PAL) and ultrasound (U) treatments for the breakdown of chlorothalonil fungicide on tomato fruit. The study involved immersing tomato fruits in PAL for 15 min and in distilled water with sonication for 15 min for individual treatments, as well as a combined treatment of PAL with sonication for 15 min. The combined treatment (PAL-U) resulted in an average reduction of chlorothalonil fungicide residues by 84.75 %. HPLC-MS analysis revealed the pathway through which chlorothalonil degrades and the formation of degradation products. Notably, these treatments did not negatively impact the quality of the tomatoes. The findings suggest that the degradation efficiency of PAL, even at reduced levels of NO_2_– and NO_3_–, can be enhanced by incorporating ultrasound treatment. This offers a promising approach for reducing fungicide residues on produce while maintaining product quality. In addition to the localized pressure and temperature impacts of ultrasound, the generation of hydroxyl radicals during cavitation plays a critical role in degrading pesticide residues when combined with other treatment techniques. This study indicates that these highly reactive hydroxyl radicals, produced as a result of ultrasound-induced cavitation, are instrumental in breaking down pesticide residues. This highlights the potential of ultrasound technology as a powerful and eco-friendly solution for eliminating pesticide residues from produce, ensuring food safety, and supporting sustainable agriculture [51].

In a related study, Ali et al. [10] investigated the potential of combining plasma-activated buffer solution (PABS) and plasma-activated water (PAW) with ultrasonication (U) treatment to reduce chlorothalonil fungicide residues while maintaining the quality of tomato fruits during storage. The combined treatment involved immersing tomato fruits in PAW and PABS, followed by 15 min of sonication. Individual treatments without sonication were also conducted for comparison. The findings of the study revealed that highest reduction of chlorothalonil fungicide residues, reaching 89.29 %, was observed in the PAW-U10 treatment. By the end of the storage period, PAW-U10 demonstrated the highest reduction rate of 97.25 %, followed by 93.14 % in PABS-U10. Notably, the application of PAW, PABS, and their combination with ultrasound did not significantly impact the overall quality of tomato fruits throughout the storage period. These findings suggest that the combination of PAW and sonication is highly effective in post-harvest agrochemical degradation, offering a promising approach to reducing fungicide residues while preserving the quality of fruits during storage.

Ahmad et al. [52] research was carried out to examine the potency of various commonly available technological treatments, including a 4 % vinegar solution and a 3 % salt solution, both with and without sonication, in reducing pesticide residues on tomato fruits. The results demonstrated that a 10-minute treatment with the 4 % vinegar solution combined with sonication had the most significant impact on pesticide reduction. Several pesticides, including Ethoprophos, Diazinon, Pirimiphos-methyl, Malathion, Penconazole, Chlorfenvinphos, Profenofos, Kresoxim-methyl, Chlorfenapyr, Diniconazole, Bifenthrin, and Fenpropathrin, were completely eliminated (100 % reduction), highlighting the treatment's effectiveness. Additionally, Atrazine and Terbufos exhibited substantial reductions of 75.13 % and 84.56 %, respectively, while pesticides such as Pirimicarb and Chlorpyrifos displayed moderate reductions of 34.52 % and 42.09 %. Notably, Pirimiphos-methyl and Malathion, among others, were completely eliminated, further emphasizing the treatment's potency in reducing pesticide residues on tomato fruits [52].

Manzoor et al. [9] reported an 80 % reduction in Anilazine fungicide in tomato juice using standalone ultrasound (40 kHz, 480 W, 40 min), driven by hydroxyl radicals and oxidative species from cavitation. Tan et al. [53] evaluated the effect of hydrogen peroxide (10 mg/L) assisted ultrasound treatment (40 kHz) on pesticide residue removal. The results indicated that this process can eliminate 90 % of Dichlorvos pesticide in tomatoes without significantly altering the vitamin C content compared to washing with water. The increased effectiveness of the ultrasonic (US) process in removing pesticide residues from vegetables is primarily due to the cavitation effect.

Yang et al. [54] applied ultrasound treatment (40 kHz) in combination with a 5 mg/L ozone solution to remove pesticide residues. The results demonstrated an average removal of over 95 % for Chlorpyrifos, Dimethoate, Carbofuran, Isoprocarb, Diniconazole, and Difenoconazole in tomatoes. The efficiency of pesticide removal during an ultrasonic (US)/ozone (O3) treatment is not only determined by the chemical properties of the pesticides but also influenced significantly by the physical characteristics of the vegetables undergoing the treatment. A study observed a distinct order of pesticide removal rates among different vegetables, with tomatoes showing higher pesticide removal than spinach, cabbage, celery, and beans. The observed variation in pesticide removal efficiency between these vegetables can be ascribed to differences in their surface properties. The specific surface area of vegetables, which is a measure of their surface area per unit mass, decreases from spinach (highest) to tomatoes (lowest), with lettuce, celery, and beans ranking in between. Vegetables with larger surface areas and rough structures allow pesticides to easily adhere to their surfaces. However, ultrasonic waves face challenges in penetrating these vegetables due to energy loss when passing through obstacles.

Incomplete pesticide degradation, as seen in cases like 15.25 % residual chlorothalonil [50] and 2.75 % in PAW-U10 [10], raises concerns about persistent residues. This can be attributed to the inherent chemical stability of certain pesticides, which resist degradation due to robust molecular structures, or to limitations in the penetration of reactive species, such as hydroxyl radicals, into complex surface matrices of produce[55]. Moreover, sonochemical degradation can generate intermediates, such as chlorpyrifos-oxon from chlorpyrifos, which may exhibit higher toxicity than the parent compound [56]. These by-products require careful monitoring through analytical techniques like HPLC-MS to ensure they do not compromise food safety. To mitigate incomplete removal and the formation of toxic intermediates, future research should focus on optimizing ultrasound parameters (e.g., frequency, power, and duration) and exploring synergistic treatment combinations to enhance degradation efficiency. Comprehensive toxicity assessments of degradation products are also essential to meet regulatory requirements and ensure the safety of ultrasound-based treatments.

### 4.1 Comparative analysis of ultrasound-based agrochemical degradation methods

Ultrasound with vinegar or hydrogen peroxide, as explored by Ahmad et al. [52] and Tan et al. [53], achieves near-complete removal (90–100 %) of pesticides like Malathion and Dichlorvos. Vinegar’s acetic acid and hydrogen peroxide’s oxidative properties enhance cavitation-induced radical formation, accelerating degradation of less stable pesticides [52]. Energy consumption is slightly lower than standalone ultrasound due to the shorter treatment time (10 min [52] compared to 40 min with ultrasound alone [9]), and the reagent cost is minimal (e.g., 4 % vinegar is widely available). Scalability is comparable to standalone ultrasound, with existing washing systems adaptable to include vinegar or peroxide solutions. However, incomplete removal of stable pesticides like Chlorpyrifos (42.09 % reduction [52]) highlights limitations, and the use of chemical additives requires wastewater treatment to remove residual acids or peroxides, increasing operational complexity. Safety concerns are minimal, as vinegar and low-concentration hydrogen peroxide are food-safe, but potential by-products (e.g., chlorinated intermediates) require monitoring to ensure non-toxicity [56]. This method is ideal for facilities seeking high efficacy with minimal equipment changes, particularly for organic produce requiring chemical-free residues.

Ultrasound with PAW or ozone, as studied by Ali et al. [10,51] and Yang et al. [54], achieves the highest reductions (84.75–97.25 % for chlorothalonil, >95 % for multiple pesticides), driven by synergistic radical generation. PAW’s reactive species (e.g., nitrates, nitrites) and ozone’s oxidative power amplify cavitation’s effects, enabling degradation of stable pesticides [10,54].

From an economic perspective, standalone ultrasound involves moderate capital costs for ultrasonic baths or flow-through systems, which are less complex than chemical treatment setups, and its operational costs are low due to short treatment times, minimal water use, and no chemical inputs, making it cost-efficient for juice processing [9]. Ultrasound with vinegar or hydrogen peroxide has similar capital costs to standalone ultrasound, as existing ultrasonic systems can incorporate low-cost reagents like vinegar or hydrogen peroxide, but operational costs are slightly higher due to reagent procurement and wastewater treatment for residual acids or peroxides [52]. Ultrasound with PAW or ozone requires significant capital investment for specialized PAW or ozone generation systems and higher operational costs due to increased energy demands and maintenance, reducing its cost-effectiveness for large-scale applications [10,54].

Standalone ultrasound preserves tomato quality by operating non-thermally, with no chemical residues, but incomplete degradation of stable pesticides raises concerns about residual toxicity [9,51]. Ultrasound with vinegar or hydrogen peroxide maintains quality, as vinegar and low-concentration hydrogen peroxide are food-safe, but potential by-products (e.g., chlorinated intermediates) require monitoring to ensure safety [57,58]. Ultrasound with PAW or ozone risks forming oxidative by-products like chlorpyrifos-oxon or aldehydes, necessitating rigorous residue and toxicity testing to meet regulatory standards, which increases operational complexity [10]. Scalability favors standalone ultrasound and ultrasound with vinegar or hydrogen peroxide, as their systems are easily integrated into existing washing lines, capable of processing large tomato volumes, facilitating rapid adoption in post-harvest facilities [52,53]. Ultrasound with PAW or ozone is less scalable due to the complexity of producing and stabilizing PAW or ozone, limiting throughput [10]. Conventional washing is scalable but less efficient due to high resource demands and variable efficacy.

Ultrasound with vinegar or hydrogen peroxide is less sustainable due to wastewater treatment needs, while ultrasound with PAW or ozone increases energy demands and system complexity, potentially offsetting environmental benefits [10]. From an industrial perspective, standalone ultrasound is the most viable for juice processing and organic produce due to its low cost, scalability, and regulatory alignment [9]. Ultrasound with vinegar or hydrogen peroxide is ideal for whole tomatoes requiring high efficacy with minimal equipment changes, while ultrasound with PAW or ozone suits specialized applications targeting stable pesticides but requires cost optimization [10,54]. Future research should focus on optimizing ultrasound parameters for recalcitrant pesticides, developing cost-effective PAW and ozone systems, and conducting comprehensive by-product analyses to ensure safety and facilitate regulatory approval.

## 5 Antimicrobial effects

**Fig. 5:**
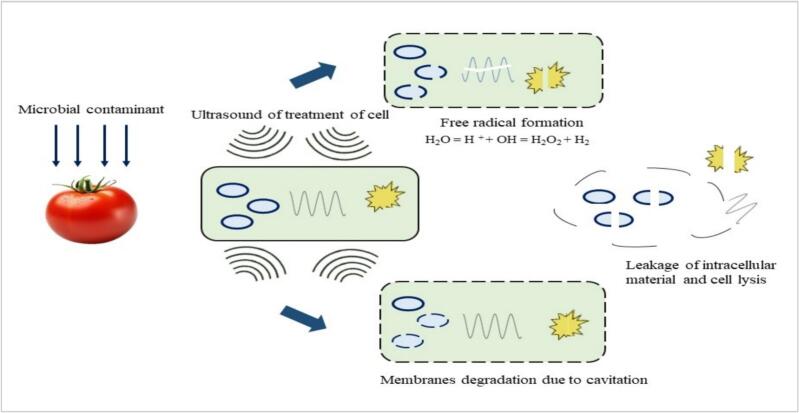
Antimicrobial effect of Ultrasound on tomato.

Pathogen-induced spoilage and seed decay pose significant challenges in tomato production, compromising yield, quality, and shelf life. Conventional thermal processing methods, such as pasteurization, often degrade nutritional content and sensory attributes, prompting the exploration of non-thermal alternatives. Conversely, non-thermal approaches, such as ultrasound, may provide effective microbial inactivation while maintaining these fresh characteristics. Ultrasound technology is widely recognized as a promising and environmentally friendly method for controlling food pathogens and preserving fresh fruits and vegetables [59]. It employs a cavitation mechanism to inactivate pathogens through mechanical disruption, localized heating, and the generation of reactive species (Fig. 5). This technology is considered safe and has been extensively utilized to inactivate pathogens and reduce microbial content in various food products [60]. This section reviews the application of ultrasound-based methods, including standalone ultrasound, ultrasound combined with chemical sanitizers (e.g., vinegar, peracetic acid, hydrogen peroxide), and multi-frequency ultrasound, in reducing microbial contamination on tomatoes. Table 4 summarizes the studies investigating the antimicrobial effects of ultrasound on tomatoes.

**Table 4:** Antimicrobial effect of Ultrasound processing on tomato.

Author & Year	Country	Parts of the tomato	Type of Processing	Type of Microorganism	Operating condition	Major Findings	Shelf-Life Extension	Ref
Mustapha et al. 2020	China	cherry tomato	US ^a^ + aqueous ozone	Mesophilic bacteria, molds & yeasts	20/40 kHz	>3 log CFU/g microbial reduction	21 days	[67]
Ding et al. 2014	China	cherry tomatoes	US + slightly acidic electrolyzed water	total aerobic bacteria, yeasts & molds	10 min	1.77 and 1.29 log reductions on total aerobic bacteria, and 1.50 and 1.29 log reductions on yeasts and molds	Not reported	[68]
Sarkinas et al. 2018	Lithuania	whole grape shaped tomatoes	US	*Listeria monocytogenes, Bacillus cereus, Escherichia coli, Salmonella typhimurium*	300 & 600 W 28 kHz, 10–30 min	The UAE has demonstrated efficacy in reducing the concentration of vegetative cells from both gram-positive and gram-negative bacteria in bacterial suspensions by 1.59 to 3.4 logarithmic units and some phyto viruses.	Not reported	[69]
Brilhante de São José et al. 2020	Brazil	Cherry tomatoes	US + alone or with 1 % lactic acid,1% detergent	*Salmonella Enteritidis*	40 kHz for 5 min	*Salmonella Enteritidis* counts (2.29 log CFU/g) in cherry tomatoes were substantially lowered to acceptable safety standards.	10 days	[35]
Zhou et al. 2023	USA	Grape tomato	US + sanitizers	*Listeria* *monocytogenes* and *Salmonella*	25 or 40 kHz 1, 2, or 5 min.	The treatment resulted in a substantial reduction of *L. monocytogenes* by 1.44–3.99 log CFU/g and a significant decrease in *S. Newport* by 1.35–3.62 log CFU/g.	Not reported	[63]
Kawakami et al. 2018	Japan	tomatoes	Aerial ultrasonic	*Fusarium oxysporum*	40.5 kHz	The application of ultrasound at frequencies of 19.8 and 28.9 kHz demonstrated a notable capacity to manage and mitigate disease	14 days	[64]
Alenyorege et al. 2018	China	tomato	multi-mode US washing treatments	Total bacteria, yeast, and mold	20–40 kHz	Reduced > 1.5 log CFU/g of bacteria, yeast, and molds	Not reported	[65]
Pinheiro et al. 2016	Portugal	Whole Tomato	US	Mesophilic, yeast and mold	45 kHz 30 min	US was not successful in significantly reducing the mesophilic bacterial counts on whole tomato fruits	30 days	[70]
Manzoor et al. 2023	China	tomato juice	US	Total viable count	40 kHz 480 W 0, 8, 16, 24, 32, and 40 min	Sonication treatment demonstrated a positive impact on the shelf life of tomato juice, as evidenced by a substantial reduction in the total viable bacterial count, decreasing from 6.31 to 1.91 log CFU/mL.	Not reported	[9]

^a^: Ultrasound.

Starek et al. [61] performed an investigation into the potential of ultrasonic technology to inactivate mesophilic aerobic microorganisms, lactic acid bacteria, coliform bacteria, and yeast while preserving the physicochemical properties of tomato juice. In the present study, pressed tomato juice was exposed to high-power ultrasound at a frequency of 20 kHz, with three key operational parameters varied to assess their influence on the outcomes: ultrasound intensity (28 and 40 W cm − 2), duration of ultrasound treatment (2, 5, and 10 min), and storage time of the treated juice (1, 4, 7, and 10 days). The efficacy of ultrasound treatment in eliminating microbial contaminants in tomato juice was found to be influenced by both the amplitude and the duration of the treatment. The study's results revealed that exposing tomato juice to an ultrasound field at an intensity of 40 W cm − 2 for 10 min effectively eradicated spoilage microorganisms, rendering the juice microbiologically pure and stable throughout a 10-day storage period. Although the ultrasonic treatment caused minor changes in lycopene content (with both increases and decreases depending on processing time) and a slight reduction in vitamin C content, it proved to be an effective method for maintaining the quality and safety of tomato juice. Similarly, De São et al. [62] investigated the efficacy of ultrasonic treatment (US) at 40 kHz for 5 min, administered individually or in combination with 1 % lactic acid (LA), 1 % commercial detergent (DET), or 6 mg/L silver nanoparticles (AgNP, with an average diameter of 100 nm), as a potential substitute for the conventional disinfectant sodium dichloroisocyanurate (200 mg/L) in inactivating Salmonella enterica serovar Enteritidis on cherry tomatoes. This research also explored the relationship between sanitizing solutions and bacterial adhesion by examining the interfacial tension between them. Findings indicated that sanitizers in solutions containing DET and AgNP exhibited lower surface tension. All treatments, except for the one with DET, effectively reduced *Salmonella Enteritidis* by more than one logarithmic cycle, with no significant difference in mean log colony-forming units (CFU)/g reduction among the various treatments.

Zhou et al. [63] examined the efficacy of various treatments, including water, chlorine, and peroxyacetic acid, either alone or combined with power ultrasound at 25 or 40 kHz for 1, 2, or 5 min, in reducing *Listeria monocytogenes* and *Salmonella Newport* on grape tomatoes. The produce was inoculated with each pathogen at 10 log CFU/g, dried for 2 h, and subsequently treated. Results revealed that the combined treatment of ultrasound and sanitizers achieved significant reductions of *L. monocytogenes* (1.44–3.99 log CFU/g) and *S. Newport* (1.35–3.62 log CFU/g) on grape tomatoes, with notably higher reductions observed compared to other produce items. The study demonstrated synergistic effects when power ultrasound was combined with chemical sanitizers, resulting in an additional 0.48–1.40 log CFU/g reduction of *S. Newport* on grape tomatoes. While these findings suggest that this combined treatment approach may not completely eliminate pathogens, it represents a valuable method for reducing bacterial contamination and enhancing food safety in the produce industry.

A study conducted by Kawakami et al. [64] demonstrated that exposure to aerial ultrasound, at a frequency of 40.5 kHz and intensity of 100 dB, for two weeks during the nursery phase could induce significant resistance to *Fusarium* wilt in tomatoes and blast disease in rice. This treatment, which ceased one week before pathogen inoculation, resulted in a marked reduction in disease incidence in both plant species. The research suggests that ultrasound irradiation may activate the salicylic acid (SA) signaling pathway, which plays a crucial role in plant defense mechanisms. This activation appears to be responsible for the reduced incidence of *Fusarium* wilt in tomato plants. The findings indicate that ultrasound irradiation could serve as a potential non-chemical method for inducing disease resistance in plants. Implementing this technique in agriculture may have significant implications for promoting sustainability by reducing reliance on chemical treatments, ultimately leading to improved crop health and yields.

Alenyorega et al. [65] investigated the effects of three different ultrasound modes: mono (20 kHz), dual (20–40 kHz), and tri-frequency ultrasound (20–40-60 kHz) on tomatoes. The study reported decreases in total plate count of 10 %, 18 %, and 35 % for MFU, DFU, and TFU, respectively. Additionally, yeast and mold counts were reduced by 11 %, 15.7 %, and 36.7 % for MFU, DFU, and TFU. These findings suggest that ultrasound, particularly at multiple frequencies, effectively reduces microbial counts on tomatoes, indicating its potential as a non-chemical method for improving food safety and extending shelf life. The observed microbial inactivation may result from the synergy of physicochemical effects during cavitation. Reactive free radicals, such as hydrogen ions (H + ) and hydroxide ions (OH–), may damage microbial cells similarly to hydroperoxides. Notably, tri-frequency ultrasound showed the most significant impact on microbial reduction, underscoring the importance of frequency combinations in ultrasound technology for food preservation [65].

Mustapha et al. [66] found that combining ultrasound waves at frequencies of 20 kHz and 40 kHz with 5 % hydrogen peroxide and peracetic acid (PAAH) led to greater reductions of aerobic mesophiles, yeasts, and molds on cherry tomatoes. The combination resulted in reductions of 3.07–3.10 log CFU/g, compared to 2.08–2.32 log CFU/g upon application of the treatment separately. Ultrasound waves exert intense pressure, facilitating the infilteration of chemical oxidants through cellular membranes. Cavitation aids in the release of microorganisms, thereby increasing the efficiency of chemical sanitizers [66].

In another study by Mustafa et al. [67], the application of dual-mode frequency irradiation (DMFI) washing treatment demonstrated a significant reduction in mesophilic bacteria (MB) and mold and yeast (M&Y) counts on the surface of fresh produce with increasing treatment time. Reductions ranged from 1.52 to 2.65 log CFU/g for MB and 1.30 to 2.56 log CFU/g for M&Y. When DMFI was combined with aqueous ozone, even more, substantial reductions were observed, with decreases of 2.09 to 3.42 log CFU/g for MB and 2.30 to 3.72 log CFU/g for M&Y. This enhanced effectiveness of ozone washing treatments may result from several factors. The cavitation caused by ultrasound weakens microbial cell walls, allowing increased penetration of ozone. Furthermore, the high pressure generated by ultrasound aids ozone permeation through cell membranes, while cavitation accelerates the collapse of microorganisms, leading to more thorough microbial elimination from the surface of produce [67].

In a study by Ding et al., [68] the combined treatment of ultrasound at 40 kHz frequency for 10 min and slightly acidic electrolyzed water on cherry tomato was observed to result in enhanced reductions of 1.77 log for aerobic bacteria and 1.5 log for yeast and mold. Ultrasonication has been shown to have the potential for inactivating bacteria in food, but it is generally not very effective at killing microorganisms at ambient or sub lethal temperatures. Sarkinas et al., [69] reported that ultrasound treatment, operating at a frequency of 28 kHz and a power of 600 W for 30 min, resulted in significant reductions in Phyto viruses in grape tomato puree and whole grape shape tomato. The Relative Optical Density of the Tobacco Mosaic Virus (TMV) decreased by 99 % in grape tomato puree and 55 % in whole grape shape tomato, which can be attributed to physical conditions. Sao José et al., [35] conducted a study comparing The utilization of ultrasound alone and in combination with various sanitizers on the microbial reduction of cherry tomatoes. The research identified that ultrasound treatment, without the addition of other sanitizers, led to a significant decrease in mesophilic aerobic bacteria by 1.3 log and a more modest reduction of coliforms by 0.27 log, which is likely due to the presence of a hydrophobic cuticle and cutin wax molecules covering the skin of the tomatoes. These molecules likely act as a barrier, limiting the effectiveness of the ultrasound treatment. Ultrasound treatment generates high pressure, which helps chemical oxidants penetrate the cell membrane. Additionally, the cavitation produced by the ultrasound aids in breaking down microorganisms, which ultimately enhances the effectiveness of the chemical sanitizer. In a separate study, Pinheiro et al., [70] evaluated the effectiveness of various preservation treatments on tomatoes, including ultrasound treatment. The results showed that ultrasound treatment, with a frequency of 45 kHz, a duration of 30 min, and a power of 80 %, resulted in a 18.9 % reduction in mesophyll load and a 21 % reduction in yeasts and mold count. Thermo Sonication treatment resulted in the greatest reduction in mesophyll counts immediately after exposure to UV-C radiation. This efficacy is likely due to the combined physical and chemical processes that occur during cavitation, which involves the formation of pores in the cell membrane, disruption of cell structures, and breakage of cells (See Fig. 5). These processes can effectively inactivate mesophyll cells, leading to a significant reduction in their count. Furthermore, several studies have explored other applications of ultrasound technology in the tomato industry such as a study investigated ultrasound's effects on the respiration rate, production of ethylene, activity of enzyme, and aromatic composition of cherry tomatoes [71]. Another study examined the influence of high-intensity ultrasound as a non-destructive physical stimulus on the levels of secondary metabolites and antioxidant capacity in tomato fruits [72]. These studies contribute valuable insights into the various ways ultrasound technology can be employed in the tomato industry. The findings highlight the importance of understanding the diverse applications and implications of ultrasound technology in the tomato industry, guiding researchers and industry professionals in developing innovative strategies for improving tomato production and processing.

### 5.1 Comparative analysis of ultrasound-based antimicrobial methods

Standalone ultrasound, ultrasound with sanitizers, multi-frequency ultrasound, and conventional thermal pasteurization each offer distinct approaches to microbial inactivation in tomato processing, with differences in efficacy, mechanisms, and costs shaping their industrial suitability. Standalone ultrasound achieves moderate microbial reductions, ranging from 0.27 to 1.3 log CFU/g for whole tomatoes, and complete eradication in tomato juice, through cavitation-induced cell disruption and hydroxyl radical generation [61,62]. However, efficacy is limited by tomato surface properties, such as the hydrophobic cuticle, which reduces cavitation penetration, particularly for whole tomatoes [61,62]. Minor nutrient losses (e.g., vitamin C [61]) require optimized parameters to balance inactivation and quality.

Ultrasound with sanitizers, such as peroxyacetic acid, chlorine, or ozone, significantly enhances reductions, achieving 1.44–3.99 log CFU/g for pathogens like Listeria and Salmonella, as sanitizers amplify cavitation’s oxidative effects and penetrate microbial membranes, ideal for whole tomatoes requiring stringent safety standards [66,67,73]. Multi-frequency ultrasound, operating at 20–60 kHz, delivers the highest reductions, with 35 % for total plate count and 36.7 % for yeasts/molds, due to intensified cavitation from multiple frequencies creating robust shear forces and reactive species, though its complexity restricts its use to specialized applications [65]. Conventional thermal pasteurization ensures near-complete microbial elimination but degrades nutrients and sensory attributes, necessitating additional processing that increases costs, particularly in quality-driven markets [28].

From an economic perspective, thermal pasteurization requires complex heat exchangers and cooling systems, leading to significant investment due to their robust design for high-temperature processing [74]. In contrast, standalone ultrasound and ultrasound with sanitizers utilize simpler ultrasonic baths or flow-through reactors, which are less costly to install and maintain, enhancing their accessibility for various facility sizes [31,61,73]. Multi-frequency ultrasound, however, involves specialized equipment with multiple transducers, resulting in higher upfront costs that may approach those of pasteurization, limiting its adoption to niche applications [65]. Operationally, thermal pasteurization incurs high costs due to substantial energy requirements for heating and cooling, as well as significant water consumption for cooling [28]. Standalone ultrasound minimizes operational costs through short treatment times, low energy requirements, and minimal water use, as it operates non-thermally without cooling needs, making it highly cost-efficient for juice processing [61]. Ultrasound with sanitizers introduces additional costs for chemicals and wastewater treatment to manage residues, but these are justified by enhanced microbial efficacy for whole tomatoes [73,75]. Multi-frequency ultrasound has higher energy demands due to multiple transducers, reducing its cost-efficiency for large-scale processing [65].

Quality preservation is a critical differentiator, as thermal pasteurization’s nutrient degradation requires fortification or reformulation, adding to costs, while ultrasound methods maintain lycopene, vitamin C, and sensory properties, reducing downstream processing needs and enhancing market value [61]. Standalone ultrasound excels in juice applications, achieving microbial safety without quality loss, while ultrasound with sanitizers supports quality preservation for whole tomatoes under stringent safety requirements [61]. Multi-frequency ultrasound’s superior efficacy benefits premium or organic produce, though its advantages are less critical for standard applications [65]. Scalability also varies, with standalone ultrasound and ultrasound with sanitizers being highly scalable, as continuous-flow systems can process large volumes of juice or whole tomatoes, facilitating faster return on investment, especially for facilities prioritizing sustainable, high-quality products [31,73]. Multi-frequency ultrasound is less scalable due to equipment complexity, while thermal pasteurization, though scalable, has a longer return on investment due to higher resource demands and quality-related expenses [65]. Ultrasound’s reduced chemical use aligns with clean-label and organic certifications, further enhancing its economic viability [28].

In summary, standalone ultrasound is the most cost-effective and scalable for tomato juice processing and low-contamination whole tomatoes, offering significant resource savings and quality preservation [61]. Ultrasound with sanitizers is optimal for whole tomatoes requiring stringent microbial control, balancing cost and efficacy [73,75]. Multi-frequency ultrasound suits premium or organic produce but is less practical for large-scale adoption due to higher costs [65]. Compared to thermal pasteurization, ultrasound methods provide comparable or superior safety with lower costs and better quality retention, making them attractive for sustainable tomato processing [28]. Future research should focus on optimizing sanitizer formulations, simplifying multi-frequency systems, and standardizing protocols to enhance cost-effectiveness and industrial applicability.

## 6 Conclusion

In conclusion, ultrasound technology presents a versatile and sustainable approach to advancing the tomato industry by enhancing bioactive compound extraction, improving seed germination, degrading agrochemical residues, and controlling microbial contamination. Its ability to significantly increase yields of valuable compounds such as lycopene and pectin while reducing processing times and solvent consumption underscores its efficiency and environmental benefits. Furthermore, ultrasound-assisted treatments effectively diminish pesticide residues and microbial loads, contributing to improved food safety and product shelf life. However, research gaps remain, including optimizing parameters for diverse tomato varieties, mitigating ROS effects, and developing standardized protocols for residue and microbial testing. Future research should optimize ultrasound frequencies and durations for specific tomato varieties, integrate it with technologies like ozone or plasma-activated water, conduct pilot-scale trials to validate scalability and develop standardized protocols for residue testing and microbial safety. By addressing these gaps, ultrasound can drive sustainable tomato processing, supporting a circular economy and food safety.

## CRediT authorship contribution statement

**Amir Shafaei Fallah:** Writing – review & editing, Writing – original draft, Investigation, Formal analysis, Data curation. **Fateme Asadi Touranlou:** Writing – review & editing. **Mitra Rezaie:** Writing – review & editing, Visualization, Validation, Supervision, Methodology, Conceptualization.

## Declaration of competing interest

The authors declare that they have no known competing financial interests or personal relationships that could have appeared to influence the work reported in this paper.
